# Transcriptional Regulation by Nrf2

**DOI:** 10.1089/ars.2017.7342

**Published:** 2018-10-24

**Authors:** Claudia Tonelli, Iok In Christine Chio, David A. Tuveson

**Affiliations:** ^1^ Cold Spring Harbor Laboratory, Cold Spring Harbor, New York.; ^2^ Lustgarten Foundation Pancreatic Cancer Research Laboratory, Cold Spring Harbor, New York.

**Keywords:** Nrf2, transcription, antioxidant response

## Abstract

***Significance:*** Nuclear factor E2-related factor 2 (Nrf2) is a transcription factor that coordinates the basal and stress-inducible activation of a vast array of cytoprotective genes. Understanding the regulation of Nrf2 activity and downstream pathways has major implications for human health.

***Recent Advances:*** Nrf2 regulates the transcription of components of the glutathione and thioredoxin antioxidant systems, as well as enzymes involved in phase I and phase II detoxification of exogenous and endogenous products, NADPH regeneration, and heme metabolism. It therefore represents a crucial regulator of the cellular defense mechanisms against xenobiotic and oxidative stress. In addition to antioxidant responses, Nrf2 is involved in other cellular processes, such as autophagy, intermediary metabolism, stem cell quiescence, and unfolded protein response. Given the wide range of processes that Nrf2 controls, its activity is tightly regulated at multiple levels. Here, we review the different modes of regulation of Nrf2 activity and the current knowledge of Nrf2-mediated transcriptional control.

***Critical Issues:*** It is now clear that Nrf2 lies at the center of a complex regulatory network. A full comprehension of the Nrf2 program will require an integrated consideration of all the different factors determining Nrf2 activity.

***Future Directions:*** Additional computational and experimental studies are needed to obtain a more dynamic global view of Nrf2-mediated gene regulation. In particular, studies comparing how the Nrf2-dependent network changes from a physiological to a pathological condition can provide insight into mechanisms of disease and instruct new treatment strategies.

## Introduction

In our everyday lives, we are continuously exposed to various chemical and physical insults, including drugs, environmental pollutants, food additives, ultraviolet and ionizing radiation. In addition to these external stresses, free radicals and reactive oxygen species (ROS) are produced as byproducts of both physiological and pathological cellular processes occurring in the mitochondria, peroxisomes, and endoplasmic reticulum (ER). At high levels, these toxicants can cause damage to cellular components, including proteins, lipids, and DNA (133). Cells normally counteract the detrimental effects of ROS and electrophiles through the activation of nuclear factor E2-related factor 2 (Nrf2; *Nfe2l2* gene name) (42). Initially identified through cloning experiments as a factor that is able to bind the nuclear factor, erythroid-derived 2/activator protein 1 (NF-E2/AP1) repeat of the beta-globin gene, Nrf2 soon became the subject of extensive research for its role in regulating the expression of many antioxidant and detoxification enzymes (58, 108, 164). Nrf2 activity and abundance are tightly regulated at the transcriptional, post-transcriptional, and post-translational level (49). In response to different activating stimuli, Nrf2 is stabilized and translocates to the nucleus, where it activates the transcription of its downstream targets (49, 117). In this review, we discuss the different modes of regulation of the Nrf2 network and highlight new emerging aspects.

## The Nrf2 Regulatory Network

### Structure of the Nrf2 protein

**FIG. 1.**
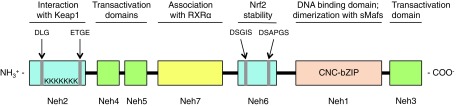
**Structure of the human Nrf2 protein.** The Nrf2 protein comprises seven Neh domains. The Neh1 CNC-bZIP domain is responsible for DNA binding and dimerization with the small Maf proteins; the Neh2 domain mediates the interaction with Keap1 through the DLG and ETGE motifs and contains seven lysine residues that are targets of ubiquitylation; the Neh3, Neh4 and Neh5 domains are transactivation domains; the Neh6 domain is a serine-rich region that regulates Nrf2 stability; and the Neh7 domain is involved in RXRα binding. bZIP, basic-region leucine zipper; CNC, cap “n” collar; Keap1, Kelch-like ECH-associated protein 1; Neh, Nrf2-ECH homology; Nrf2, nuclear factor E2-related factor 2; RXRα, retinoid X receptor α.

Nrf2 (59, 108) belongs to the cap “n” collar (CNC) subfamily of basic-region leucine zipper (bZIP) transcription factors together with Nrf1 (18), Nrf3 (79), NF-E2 p45 subunit (7), as well as the more distantly related factors BTB domain and CNC homolog 1 and 2 (Bach1 and Bach2) (127). Nrf2 is a modular protein with seven Nrf2-ECH homology domains (Neh1–7), each of which fulfills distinct functions (Fig. 1) (49). The Neh1 domain comprises the CNC-bZIP region that is necessary for DNA binding and association with Nrf2 dimerization partners, the small masculoaponeurotic fibrosarcoma (sMaf) proteins (110). Of note, the amino acid sequence of this domain, in particular the basic region, is highly conserved across a wide range of species, underlining how crucial the transcriptional activity is for Nrf2 function (38). The Neh2 domain contains two highly conserved amino acid stretches, the DLG and ETGE motifs, which mediate the interaction with Nrf2 negative regulator Kelch-like ECH-associated protein 1 (Keap1) and seven lysine residues targeted for ubiquitylation and subsequent proteasomal degradation of Nrf2 (60, 102, 166). The C-terminal Neh3 domain harbors transactivation activity and functions in concert with the Neh4 and Neh5 domains to activate transcription of Nrf2 target genes (71, 120, 143). The Neh6 domain is a serine-rich region that is involved in the negative regulation of Nrf2 stability independent of Keap1. It contains two conserved peptide motifs, DSGIS and DSAPGS, which are recognized by β-transducing repeat-containing protein (β-TrCP) (24). β-TrCP binds more efficiently to the Neh6 domain after glycogen synthase kinase-3β (Gsk-3β)-mediated phosphorylation of the DSGIS motif and promotes the recruitment of Skp1-Cul1-F-box protein (SCF) ubiquitin ligase complex and consequent proteasomal degradation of Nrf2 (131, 132, 158, 183). The Neh7 domain is involved in the repression of Nrf2 transcriptional activity by the retinoid X receptor α through a physical association between the two proteins (175).

### The Nrf2-Keap1-ARE stress–response pathway

**FIG. 2.**
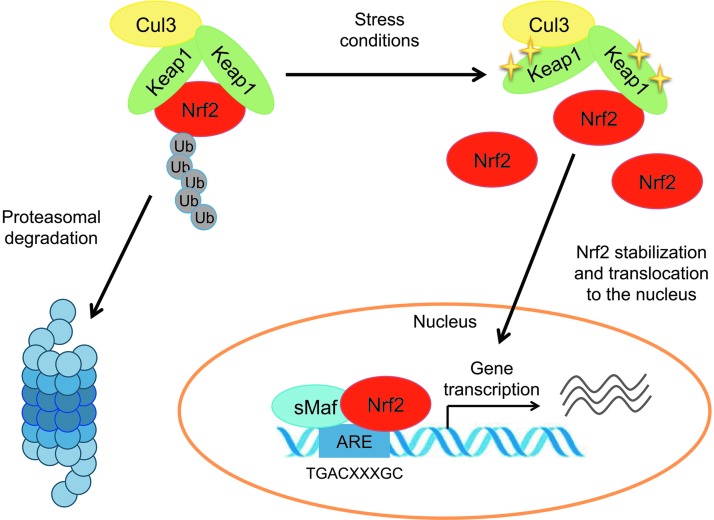
**The classical view of Nrf2 activation and response.** Under unstressed conditions, Nrf2 is bound to Keap1, constantly ubiquitylated by the Cul3 E3 ubiquitin ligase and subsequently degraded by the proteasome. In response to stress, Keap1 is inactivated, resulting in Nrf2 stabilization. Nrf2 translocates to the nucleus where it heterodimerizes with the small Maf proteins, binds to the ARE and activates the transcription of its target genes. ARE, antioxidant response element.

Nrf2 abundance within the cell is tightly regulated by Keap1, a redox-sensitive E3 ubiquitin ligase substrate adaptor (60). Keap1 was initially identified in a yeast two-hybrid screen by using the Neh2 domain of Nrf2 as bait and was confirmed as an Nrf2 repressor by showing that mouse embryonic fibroblasts (MEFs) and livers from *Keap1* knockout mice express constitutively high levels of Nrf2 and Nrf2 target genes (60, 171). Under homeostatic conditions, two molecules of Keap1 are bound to the Neh2 domain of Nrf2 at the ETGE and DLG motifs (*via* their Kelch-repeat domain) (Fig. 2) (166). Keap1 functions as an adaptor protein for the Cul3 E3 ubiquitin ligase, which is responsible for the continuous ubiquitylation and degradation of Nrf2 (28, 37, 80, 192). Under unperturbed conditions, Nrf2 has a short half-life of approximately 10–30 min; therefore, Keap1-mediated high turnover of Nrf2 keeps Nrf2 basal levels extremely low (118, 156). In response to oxidative stress, Keap1 is oxidized at reactive cysteine residues, resulting in Keap1 inactivation, Nrf2 stabilization and translocation into the nucleus (10, 32, 61, 92). Here, Nrf2 heterodimerizes with members of the sMaf protein family (MafF, MafG, and MafK) (112). Genetic evidence for the essential role of sMaf proteins as Nrf2 binding partners came from crossing *sMaf* knockout mice to *Keap1* knockout mice. *Keap1*-null animals show postnatal lethality due to hyperactivation of Nrf2, which results in aberrant proliferation of keratinocytes in the esophagus and forestomach (171). This phenotype is reversed not only by the concomitant disruption of *Nfe2l2* (171) but also by the simultaneous deletion of both *MafG* and *MafF*, thus identifying the sMafs as essential Nrf2 dimerization partners (110).

The Nrf2-sMaf complex binds, in a sequence-specific manner, to the antioxidant response element (ARE 5′-TGACXXXGC-3′) in the promoter region of Nrf2 target genes. The AREs were initially identified as *cis*-regulatory elements for *NADPH quinone dehydrogenase 1* (*Nqo1*) and *glutathione S-transferase* (*Gst*) genes (36, 136, 161). Subsequent studies expanded the list of proteins that are encoded by the ARE gene battery including genes involved in drug detoxification, antioxidant responses, NADPH regeneration and regulation of metabolism (49). *Nfe2l2* knockout mice provided *in vivo* evidence that Nrf2 regulates the expression of these antioxidant and cytoprotective genes (58, 164).

### Nrf2-mediated response to xenobiotic and oxidative stress

**FIG. 3.**
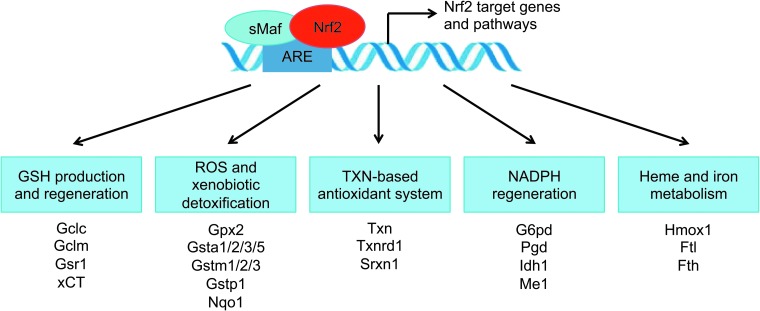
**The Nrf2-regulated cytoprotective defense system.** Through the coordinated regulation of GSH and TXN production, utilization and regeneration, NADPH regeneration, heme and iron metabolism, ROS and xenobiotic detoxification, Nrf2 provides the main cytoprotective defense system in the cell. GSH, glutathione; HMOX1, heme oxygenase 1; Idh1, isocitrate dehydrogenase 1; NAPDH, nicotinamide adenine dinucleotide phosphate; Nqo1, NADPH quinone dehydrogenase 1; Pgd, 6-phosphogluconate dehydrogenase; ROS, reactive oxygen species; TXN, thioredoxin.

Nrf2 controls the expression of key components of the glutathione (GSH) and thioredoxin (TXN) antioxidant system, as well as enzymes involved in NADPH regeneration, ROS and xenobiotic detoxification, heme metabolism, thus playing a fundamental role in maintaining the redox homeostasis of the cell (Fig. 3) (42). Nrf2 tightly regulates GSH levels by directly controlling the expression of the two subunits that constitute the glutamate-cysteine ligase (Gcl) complex: the catalytic subunit (Gclc) and the modifier subunit (Gclm) (109, 180). Gcl catalyzes the reaction of glutamate with cysteine, the rate-limiting step in the synthesis of GSH. Cysteine is generated from the reduction of cystine, which is imported into the cell by the system x_c_^−^ (11, 22). Nrf2 increases the supply of cysteine by directly activating *Slc7a11*, the gene encoding the xCT subunit of system x_c_^−^ (141). In addition to GSH synthesis, Nrf2 plays a role in GSH maintenance. Nrf2 regulates the transcription of numerous ROS-detoxifying enzymes such as glutathione peroxidase 2 (Gpx2) and several glutathione S-transferases (Gsts) (Gsta1, Gsta2, Gsta3, Gsta5, Gstm1, Gstm2, Gstm3 and Gstp1) (19, 164). These enzymes use GSH to inactivate ROS, generating oxidized glutathione (GSSG). GSSG is reduced back to GSH by glutathione reductase 1 (Gsr1), another Nrf2 target, in an NADPH-dependent manner (46). Through the coordinated activation of GSH production, utilization, and regeneration, Nrf2 ensures that intracellular levels of reduced GSH are maintained. In addition to the regulation of GSH levels within the cells, Nrf2 controls the thioredoxin (TXN)-based antioxidant system. Nrf2 regulates the expression of TXN (48), thioredoxin reductase 1 (Txnrd1) (139, 170), and sulfiredoxin (Srxn1) (1), which are essential for the reduction of oxidized protein thiols (48).

NADPH is an obligatory cofactor for many drug-metabolizing enzymes and antioxidant systems, such as cytochromes p450 (Cyp) enzymes and the Nrf2 target Nqo1 (49). Nrf2 supports NADPH production through the positive regulation of the principal NADPH-generating enzymes: glucose-6-phosphate dehydrogenase (G6pd), 6-phosphogluconate dehydrogenase (Pgd), isocitrate dehydrogenase 1 (Idh1), and malic enzyme 1 (Me1), as shown in primary cortical astrocytes (91), lung cancer cells (107), mouse small intestine (164), and mouse liver (184). Another important cytoprotective enzyme regulated by Nrf2 is heme oxygenase (Hmox1), which catalyzes the breakdown of heme molecules (5). Heme degradation results in the release of free Fe^2+^. Fe^2+^ catalyzes the Fenton reaction, which describes the conversion of H_2_O_2_ to the highly damaging OH^•^ radical (43). To prevent OH^•^ formation, in conjunction with *Hmox1* upregulation, Nrf2 induces the expression of the genes encoding the components of the ferritin complex: the ferritin light polypeptides (Ftl) and heavy polypeptides (Fth) (23, 184). The ferritin complex oxidizes Fe^2+^ to Fe^3+^ and stores it within its own structure, thus making it unavailable for the Fenton reaction (126). Additionally, Nrf2 can also influence cellular elimination of xenobiotics by controlling the expression of many phase I and phase II drug-metabolizing enzymes (49), as well as the multi-drug-resistance-associated transporters (Mrps) (99, 188). In summary, Nrf2 increases the cellular defense mechanisms against xenobiotic and oxidative stress through the coordinated expression of numerous antioxidant and detoxification genes. The Nrf2 cytoprotective response has been elucidated in various mammalian tissues and cultured cells. In addition, other model organisms, such as *Danio rerio*, *Drosophila melanogaster*, and *Caenorhabditis elegans*, have been shown to possess similar anti-stress systems to mammals, suggesting that the Nrf2 antioxidant system represents an evolutionary conserved defense mechanism (38). Of note, *C. elegans* does not have an authentic ortholog of Keap1 (14). Skinhead-1 (Skn-1), the Nrf2 ortholog, seems to be regulated at the protein level; however, the mechanism is unclear (38). This suggests that the redox-sensing function of Keap1 might have been acquired later during evolution.

### Emerging functions of Nrf2

In recent years, additional functions of Nrf2 have been discovered that go beyond the classical view of Nrf2 as a master regulator of antioxidant responses. For example, Nrf2 has been shown to regulate mitochondrial bioenergetics (54). In murine neurons and embryonic fibroblasts, loss of Nrf2 decreases the mitochondrial membrane potential, ATP production and respiration (54). In agreement with this observation, mitochondrial oxidation of the long-chain palmitic acid and the short-chain hexanoic acid is diminished in *Nfe2l2* knockout MEFs (96). In addition, Nrf2 has been shown to be involved in the unfolded protein response (UPR), which is triggered by the accumulation of misfolded proteins in the ER lumen (104, 176). In the absence of Nrf2, several UPR-associated proteins show reduced expression in the liver of mice on a high-fat diet (104). Furthermore, Nrf2 can promote the removal of damaged or misfolded proteins by regulating proteasome activity (69, 87, 88). Multiple proteasome subunits are upregulated after treatment with Nrf2 inducers both in mouse tissues and in human fibroblasts (69, 87, 88). Nrf2 can therefore prevent the accumulation of abnormal proteins that might otherwise interfere with cellular functions (69, 87, 88, 104).

Moreover, Nrf2 has been shown to regulate intermediary metabolism (29, 107). In human lung cancer cells, NRF2 regulates serine biosynthesis *via* activating transcription factor 4 (ATF4) and phosphoglycerate dehydrogenase (PHGDH) to support GSH and nucleotide production and coordinately activates the pentose phosphate pathway (PPP) to supply ribose for nucleic acid biosynthesis (29, 107). In proliferating cells, the oxidative PPP and serine-driven one-carbon metabolism are the main contributors to cytosolic NADPH production (35). By controlling the PPP and thus the fluctuations in NADPH levels that affect the oxidation of peroxiredoxin, Nrf2 has been shown to influence the transcriptional oscillations of the circadian genes in human cells, mouse tissues and living flies (134). This provides an example of how Nrf2 can influence other cellular processes indirectly, through the regulation of redox homeostasis. In normal stem cells, Nrf2-mediated redox control plays an important role in maintaining stem cell quiescence (53, 63, 130, 167). In *Drosophila* intestinal stem cells (ISCs), constitutive activation of cap ‘n’ collar isoform C (CncC), a homolog of Nrf2, sustains quiescence by maintaining low intracellular ROS levels (53). In response to paraquat-induced stress, Keap1-mediated repression of CncC results in the accumulation of ROS that promotes ISCs proliferation and accelerates age-related degeneration of the intestinal epithelium (53). In ISCs, the regulation of CncC works in the opposite way compared to differentiated cells: CncC activity is inhibited in response to stress in ISCs, whereas it is induced in differentiated cells. The mechanism of CncC repression in ISCs is still unclear, but it involves Keap1 (53). Similar to ISCs, low intracellular ROS levels are required for the maintenance of quiescence in mouse and human airway basal stem cells (ABSCs) (130). After polidocanol-mediated injury of the mouse tracheobronchial epithelium, changes in ROS levels from low to moderate activate Nrf2, which induces the Notch pathway to stimulate stem cell self-renewal and proliferation for repair (130). In parallel, Nrf2 induces antioxidant genes that return overall ROS levels to a low state and this inhibits ABSCs proliferation, thus preventing hyperproliferation that would be detrimental for the repairing tissue (130). Nrf2 also regulates proliferation and differentiation of mouse hematopoietic stem cells under physiological conditions (167). Nrf2 controls the expansion of the stem and progenitor cells and supports their efficient homing by positively regulating C-X-C chemokine receptor type 4 (Cxcr4) (167). In addition to stem cell self-renewal, NRF2 participates in human embryonic stem cells differentiation into neuroectoderm (63). These studies provide examples of how redox control by Nrf2 can affect different physiological processes through the regulation of ROS levels. It is however unclear which are the targets of ROS cytotoxicity that would explain the observed phenotypes. In this regard, some light has been shed in pancreatic cancer, where Nrf2 antioxidant activity is known to be important for tumor initiation and progression (30). Cysteine residues contain highly reactive thiol groups that render them sensitive to changes in intracellular ROS levels. Alterations in the cellular redox levels are therefore likely to affect the oxidation status of reactive cysteine-containing proteins. The development of a highly sensitive proteomic method to quantify changes in the cysteine proteome showed that Nrf2-antioxidant activity promotes pancreatic tumor maintenance by preventing cysteine oxidation of the mRNA translational machinery to support efficient protein synthesis (21). This cysteine proteomic approach can now be applied to investigate the impact of changes in ROS levels on additional cellular processes.

## Modes of Regulation of Nrf2 Activity

**FIG. 4.**
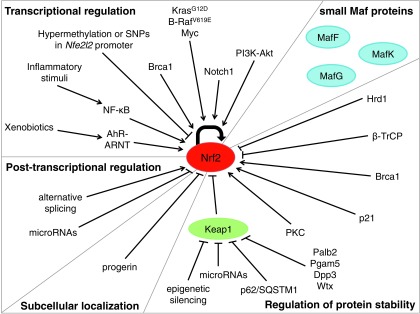
**Mechanisms of regulation of Nrf2 activity.** The mechanisms of modulation of Nrf2 activity include the regulation of transcription, mRNA processing, translation, subcellular localization, protein stability, and availability of binding partners. *Arrows* indicate activating regulation, and *bars* indicate inhibitory regulation. β-TrCP, β-transducing repeat-containing protein; AhR, aryl hydrocarbon receptor; BRCA1, breast cancer susceptibility 1; NF-κB, nuclear factor-κB; PI3K, phopshoinositide 3-kinase; PKC, protein kinase C.

Given the vast array of stimuli that activate Nrf2 and the diverse cellular processes that it controls, the regulation of Nrf2 activity is complex and multifactorial. Indeed, Nrf2 activation can be controlled at the transcriptional and post-transcriptional level, through the regulation of protein stability, post-transcriptional modifications, and the availability of binding partners (Fig. 4).

### Transcription-associated regulation of Nrf2

*Nfe2l2* transcription is regulated by several transcription factors. Nrf2 is induced by aryl hydrocarbon receptor (AhR) in response to polycyclic aromatic hydrocarbon exposure (106). AhR binds as a heterodimer with AhR nuclear translocator (Arnt) to the xenobiotic response element-like sequences in the promoter of *Nfe2l2* and transactivates its transcription (106). Thus, xenobiotic ligands are able to activate the Nrf2 pathway by inducing AhR. In addition, the *NFE2L2* promoter contains a binding site for nuclear factor (NF)-κB, which allows it to be induced by inflammatory stimuli (138). *NFE2L2* transcription is indeed activated by lipopolysaccharide (LPS) treatment in human monocytes (137). High basal NRF2 activity in acute myeloid leukemia (AML) has been attributed to constitutive NF-κB-mediated upregulation of the *NFE2L2* gene and is believed to be one cause of resistance to chemotherapy in AML cells (138). In addition, *NFE2L2* is induced by breast cancer susceptibility 1 (BRCA1) in human MCF10A mammary epithelial cells on xenobiotic stress (67). Oncogenic Kras and B-Raf, Myc (30), the phosphoinositide 3-kinase (PI3K)-Akt pathway (107), and the Notch signaling pathway (173) have also been reported to augment *Nfe2l2* transcription, thus suggesting a possible mechanism for the increased expression of Nrf2 in tumor cells. Of note, the *Nfe2l2* gene promoter contains ARE-like sequences, providing a positive feedback mechanism to amplify Nrf2 effects (86). Indeed, in murine keratinocytes, Nrf2 has been shown to bind these sequences and overexpression of the wild-type but not a truncated form of Nrf2 lacking the N-terminal region (amino acids 1–368, including the transactivation domains) induces the activity of the isolated promoter-proximal region of the *Nfe2l2* gene in a luciferase reporter assay (86). Additional evidence for the transcriptional regulation of Nrf2 came from the observation that modifications of the *Nfe2l2* promoter region such as hypermethylation or single nucleotide polymorphisms (SNPs) result in decreased Nrf2 expression; however, the significance of these alterations awaits further study (101, 159, 191).

### Post-transcriptional regulation of Nrf2

microRNAs (miRNAs) are short (20–22 nucleotides long), single-stranded, noncoding RNAs that regulate gene expression by sequence-specific binding with mRNA molecules and consequent inhibition of translation or degradation of the targets (181). miR-144 was the first miRNA identified as an NRF2 negative regulator in reticulocytes of patients with homozygous sickle cell disease (HbSS) (140). A subset of HbSS patients with more severe anemia shows higher erythrocytic miR-144 expression. Increased miR-144 is associated with reduced NRF2 levels, decreased GSH regeneration and impaired oxidative stress tolerance, thereby providing a possible mechanism for the increased anemia severity seen in these patients (140). Subsequently, other miRNAs have been identified to control Nrf2 levels in the cell. Ectopic expression of miR-28 in human MCF7 breast cancer cells (187); miR-27a, miR142-5p, miR-144, and miR-153 in human SH-SY5Y neuroblastoma cells (116); and miR-93 in human MCF10A mammary epithelial cells and T47D breast cancer cells decreased NRF2 mRNA and protein levels (151). However, validation in physiological conditions is still lacking.

Nrf2 can also be regulated through alternative splicing. In lung and head and neck cancers, aberrant *NFE2L2* transcript variants missing exon 2, or exons 2 and 3, have been observed (40). The NRF2 protein isoforms encoded by these splice variants lack the KEAP1 interaction domain, thus resulting in NRF2 stabilization and induction of the NRF2 program (40). The impact of *NFE2L2* exon skipping on tumorigenesis remains to be evaluated. However, silencing of NRF2 in a hepatocellular carcinoma cell line with heterozygous skip of *NFE2L2* exon 2 results in decreased cell viability (40).

### Regulation of Nrf2 protein stability

Under unstressed conditions, Nrf2 is constantly targeted for proteasomal degradation by the Keap1*/*Cul3 E3 ubiquitin ligase complex (37, 60, 80, 192). The high turnover of Nrf2 provides a readily available pool of newly translated protein that can be rapidly stabilized in response to stress by inhibiting ubiquitylation and proteasomal degradation. A decrease in the amount of Keap1 results in Nrf2 accumulation, as evidenced by deletion of *Keap1* in the mouse and knockdown of *Keap1* in human cells (31, 171). Epigenetic silencing of *KEAP1* by hypermethylation of its promoter causes an increase in Nrf2 expression levels in lung (114, 177), prostate (194), colorectal cancers (45) and gliomas (113), conferring a growth advantage to the cancer cells. In addition, *KEAP1* is negatively regulated by miR-200a in breast cancer cells, leading to increased NRF2 stabilization (33).

The interaction between NRF2 and KEAP1 can also be disrupted by somatic mutations of the *NFE2L2* and *KEAP1* genes, as observed in carcinomas of the lung (123, 147, 150, 153, 190), gallbladder (146), ovary (84), breast (119, 152), stomach, liver (190), skin, larynx, and esophagus (77). Moreover, several cytoplasmic proteins that interfere with Keap1-Nrf2 interaction have been identified. These include p62, also known as sequestosome 1 (SQSTM1), a ubiquitin-binding protein that targets protein aggregates for degradation *via* the autophagic pathway (26, 76, 83, 90). The STGE motif of p62 is similar to the ETGE motif of Nrf2 and therefore p62 competes with Nrf2 for the binding with Keap1. When autophagy is impaired, p62 levels are elevated, leading to degradation of Keap1 and consequent Nrf2 stabilization (26, 83, 90). Of note, p62 is an Nrf2 target gene, thus creating another positive feedback loop (62). In addition to p62, other proteins that bind Keap1 and interfere with Keap1-Nrf2 interaction include partner and localizer of Brca2 (PALB2) (97), phosphoglycerate mutase 5 (PGAM5) (95), dipeptidyl-peptidase 3 (DPP3) (47), and Wilms tumor gene on X chromosome (WTX) (16), among others. Competing proteins that disrupt the Keap1-Nrf2 association by physically interacting with Nrf2 have also been identified. The cyclin-dependent kinase inhibitor p21 is induced by p53 in response to oxidative stress and competes with Keap1 for the binding to the DLG motif of Nrf2, thus compromising Nrf2 ubiquitylation and promoting the Nrf2-dependent antioxidant response (20, 34). The DNA repair protein Brca1 can also induce Nrf2 stabilization by binding Nrf2 and preventing Keap1-mediated inhibition (41). Loss of Brca1 in mouse premalignant mammary epithelial cells results in reduced expression of Nrf2 and Nrf2-regulated antioxidant enzymes, leading to accumulation of ROS (41).

Additionally, Keap1-mediated regulation of Nrf2 activity can be modulated by post-translational modifications of Nrf2. Protein kinase C (PKC) phosphorylates Ser40 in the Neh2 domain of NRF2, disrupting KEAP1-NRF2 association and thus promoting NRF2 activation (55). This allows Nrf2 activity to be induced by signals that induce PKC, such as oxidative stress (155).

Keap1 is not the only negative regulator of Nrf2. Keap1-independent degradation of Nrf2 was first noted when it was observed that deletion of the ETGE and/or DLG motifs in the Neh2 domain results only in a modest increase in its stability under unstressed conditions (103). Further examination of the Nrf2 protein sequence led to the identification of two highly conserved regions within the Neh6 domain of Nrf2, deletion of either of which increases the half-life of Nrf2 mutants that lack the Neh2 domain in *Keap1*-null MEFs (24). These regions contain two peptide sequences, the DSGIS and the DSAPGS motifs, which are recognized by β-TrCP. β-TrCP binding to Nrf2 follows Gsk-3β-mediated phosphorylation of the Neh6 domain of Nrf2. β-TrCP functions as an adaptor for the SCF E3 ubiquitin ligase complex to regulate proteasomal degradation of Nrf2 (131, 132). Thus, Nrf2 activity could potentially be enhanced by some kinases, such as extracellular signal-regulated kinase (ERK), p38 MAP kinase (MAPK), PI3K and PKC, through the inhibition of Gsk-3β (66). Another ubiquitin-dependent system responsible for Nrf2 degradation involves the E3 ubiquitin ligase synoviolin (Hrd1) (185). During ER stress in the context of liver cirrhosis induced experimentally by administration of CCl_4_, the Nrf2 antioxidant activity is repressed by Hrd1. Hrd1 interacts with the Neh4 and Neh5 domains of Nrf2 though its C-terminal domain and causes Nrf2 ubiquitylation and subsequent degradation. Hrd1-mediated regulation of Nrf2 is independent of both Keap1 and β-TrCP and prevents Nrf2 from activating the antioxidant response and therefore counteracting the high levels of ROS produced during cirrhosis. Thus, pharmacological inhibition of Hrd1 may represent a potential therapeutic strategy for mitigating liver cirrhosis (185).

### Regulation of Nrf2 subcellular localization

Hutchinson-Gilford progeria syndrome (HGPS) is a rare, invariably fatal genetic condition characterized by premature aging beginning in childhood (169). The disease is caused by constitutive production of progerin, a mutant form of the nuclear architectural protein lamin A (39). HGPS cells present a variety of cellular defects including nuclear distortion, loss of heterochromatin structure, altered patterns of histone modifications and increased levels of persistent DNA damage (39). Until a few years ago, the mechanism through which progerin caused these morphological and epigenetic alterations was unclear. Recently, it was shown that progerin traps NRF2 at the nuclear periphery, thus impairing its activity (85). NRF2 sequestration by progerin results in chronic oxidative stress and contributes to HGPS aging defects, which can be reverted by the reactivation of NRF2. Thus, impairment of NRF2 activity is a driver mechanism of HGPS and restoration of its function may represent a therapeutic opportunity for HGPS patients (85).

### Small Maf proteins

The regulation of Nrf2 activity is not limited to the control of its abundance but can also be modulated by the availability of its binding partners. As mentioned earlier, Nrf2 forms heterodimers with the sMaf proteins and recognizes the AREs in the genome (72). The sMafs (MafF, MafG and MafK) are members of the bZIP family of transcription factors: the basic domain binds DNA, whereas the leucine zipper mediates homo- or hetero-dimerization with CNC proteins and Bach proteins (68). The CNC and Bach proteins cannot bind DNA on their own and require sMafs as obligatory dimerization partners to exert their role as transcriptional regulators (112, 127). The sMaf proteins lack the transactivation domain and sMaf homodimers have been shown to act as transcriptional repressors in overexpression experiments (56, 78, 115). Thus, the sMaf proteins and their binding partners form a complex network of interacting transcription factors. Changes in the abundance or activity of the participant molecules of the network can lead to major changes in the regulation of gene expression (111).

*sMaf* genes expression is detected in various tissues, but each *sMaf* gene has a distinct expression profile (124, 165). In human adult tissues, *MAFK* mRNA levels are high in heart, skeletal muscle, and placenta; whereas *MAFG* mRNA is abundant in skeletal muscle and is moderately expressed in heart and brain. Both are expressed in all hematopoietic cell lines, including erythroid and megakaryocytic lineages (165). In the mouse, *MafK* and *MafG* are expressed in most tissues, albeit at different levels, whereas *MafF* gene expression is restricted to the lung (124). The sMaf proteins show a high degree of conservation among vertebrates, including human, mouse, rat, chicken, and zebrafish, and a significant similarity in the primary structure to each other (68, 74). The high degree of similarity suggests that the sMaf proteins have redundant activity and that the specificity is determined by their expression pattern.

To dissect *in vivo* the specific functions of the different sMafs, *MafF*, *MafG*, and *MafK* knockout mice were generated by deleting the entire coding sequence (124, 125, 145). Gene targeting of *MafF* and *MafK* does not cause any apparent phenotype (124, 125), while *MafG*-null mice exhibit abnormal megakaryocyte differentiation and thrombocytopenia accompanied by a late-onset neurological disorder (145). *MafK* and *MafG* double knockout mice survive embryogenesis, but they die postnatally (125). These mutant mice develop more severe deficiencies in megakaryopoiesis compared with *MafG*-null mice, specifically in proplatelet formation, resulting in profound thrombocytopenia (125). In addition, they present severe anemia accompanied by abnormal erythrocyte morphology and develop severe neurological disorders (73, 125). These observations indicate that MafG and MafK have redundant functions, although MafG is preponderant (73, 125). Mice deficient for *Nfe2*, another member of the CNC subfamily of bZIP transcription factors (7), exhibit impaired megakaryopoiesis, suggesting that the NF-E2 p45-sMafG heterodimer is necessary for the production of platelets from megakaryocytes (149). Mice carrying a central nervous system-specific deletion of *Nfe2l1*, the gene encoding Nrf1, display similar neurological disorders as the ones observed in *sMaf*-deficient mice, indicating that Nrf1 and the sMaf proteins likely collaborate in maintaining neuronal homeostasis (81).

*MafF*, *MafG*, and *MafK* triple knockout embryos develop normally until embryonic day 9.5 (E9.5), then show severe growth retardation and liver hypoplasia and die around E13.5 (186). Basal expression of ARE-dependent genes is unaffected in E10.5 triple knockout embryos compared to wild-type embryos but is significantly reduced in the livers of E13.5 mutant embryos in concomitance with the severe liver hypoplasia observed in these embryos (186). Importantly, the embryonic lethality and liver hypoplasia could be completely rescued by transgenic expression of exogenous MafG (186). Basal expression of cytoprotective genes is severely compromised in *sMaf* triple knockout fibroblasts prepared from E11 or E13.5 embryos, confirming that the sMafs are essential for the expression of ARE-regulated genes (72). MafG is sufficient to rescue the inducible expression of cytoprotective genes in MEFs (72).

In summary, the investigation of *sMaf* knockout mice showed that the sMaf proteins are functionally redundant and indispensable for supporting Nrf2-mediated transcriptional activity (72, 186).

## Mechanisms of Nrf2-Mediated Gene Transactivation

**FIG. 5.**
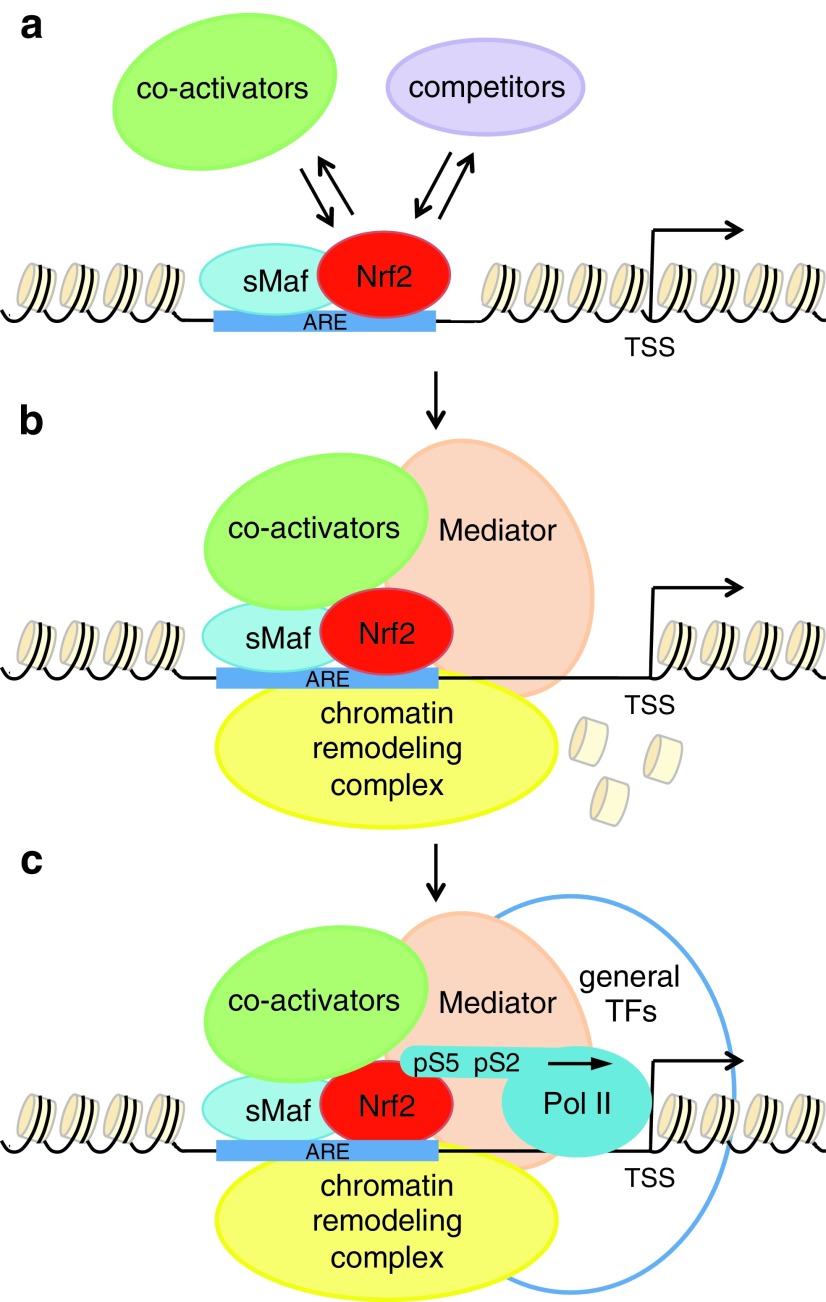
**Nrf2-mediated induction of gene expression. (a)** Nrf2 selects the genes to be activated by binding as a heterodimer with sMaf to the ARE in promoter regions of the target genes. The recognition of the ARE can be influenced by the cooperation or competition with other activators or repressors. **(b)** Nrf2 recruits co-activators, components of the transcription machinery and nucleosome-remodeling complexes through protein–protein interactions to make the chromatin structure accessible to the Pol II machinery. **(c)** Together, co-activators and chromatin remodelers favor the recruitment of Pol II and the general transcription factors to form the PIC. Following phosphorylation of Ser2 and Ser5 in Pol II CTD transcription starts. CTD, carboxy-terminal domain; PIC, pre-initiation complex; Pol II, RNA polymerase II; sMaf, small masculoaponeurotic fibrosarcoma.

Nrf2 activity is tightly regulated (160). Once Nrf2 is activated, it translocates to the nucleus where it binds to target sequences in association with the sMaf proteins (Fig. 5) (49). The investigation of Nrf2-DNA interactions in a genome-wide manner through chromatin immunoprecipitation followed by sequencing (ChIP-Seq) in MEFs from *Keap1*-null mice showed that the ARE is strongly enriched within Nrf2 binding sites (100). Subsequent ChIP-Seq analyses of Nrf2 binding sites in human lymphoblastoid cells treated with the dietary isothiocyanate sulforaphane (SFN) and in the mouse hepatoma cell line Hepa1c1c7 treated with the electrophilic agent diethyl maleate (DEM) showed preferential binding of Nrf2-sMaf heterodimer to 5′-TGACTCAGC-3′ (23, 52). In addition, these studies showed that a small fraction of Nrf2 binding sites did not contain an ARE, indicating that Nrf2 probably interacts with other DNA-binding proteins (23, 52, 100). However, the functional relevance of ARE-independent binding requires further investigation.

The extent of Nrf2 transactivation depends on the levels of Nrf2 protein, as shown in a gene dose response study analyzing expression changes in livers from *Nfe2l2*-null, wild-type, *Keap1* knockdown and *Keap1* knockout mice (184). Genes involved in the antioxidant response, GSH and xenobiotic metabolism show a graded activation by Nrf2, suggesting that the Nrf2-regulated cytoprotective response can be tuned to the intensity of the stress by varying Nrf2 levels (184).

The integrated analysis of Nrf2 binding and transcription profiles showed that not all genes in the vicinity of bound Nrf2 are transcriptionally regulated as a result of Nrf2 binding (23, 52, 100). These genes may require the recruitment of specific cofactors for a complete activation. Motif analysis of Nrf2 binding sites identified the consensus motifs for other transcription factors, such as Fos, Mafb, Lhx3 and MEF2A; however, further experiments are required to evaluate their cooperation with Nrf2 to induce gene transactivation (100). In addition, ARE-like sequences are recognized by other CNC transcription factors and the members of the AP1 complex, such as Jun, Fos, Atf and Maf proteins in electrophoretic mobility shift assays (4) and NRF2 was reported to form heterodimers with ATF4 (50). Full understanding of Nrf2-mediated gene transactivation requires taking into account the cooperation or competition with other transcription factors at the available binding sites.

Once the decision to activate a gene is made, Nrf2 recruits co-activators and components of the transcription machinery through protein–protein interactions to initiate transcription. One of the first co-activator identified to interact with Nrf2 is CREB binding protein (CBP) (25, 71). Nrf2 binds CBP through its Neh4 and Neh5 domains and together they activate transcription *via* the AREs (71). CBP can stimulate gene transcription through its histone acetyltransferase activity or by functioning as a scaffold to stabilize additional components of the general transcriptional machinery (12, 17). However, the precise mechanism of how Nrf2 and CBP cooperate to transduce the ARE gene battery remains to be elucidated (71). In addition to CBP, NRF2 interacts with its close homologue p300 (143). As CBP, p300 acetylates histones to facilitate chromatin decondensation and the recruitment of the transcription machinery (122). In addition, p300/CBP have been reported to associate with NRF2 in response to arsenite-induced stress and acetylate a number of lysine residues within the Neh1 DNA binding domain of NRF2 (157). Mutation of 18 lysine sites to arginine does not affect NRF2 protein stability but does compromise the DNA-binding ability of NRF2 and consequently the transcription of *NQO1*, *TXNRD1* and *GCLM*, but not *HMOX1* in HCT116 cells (157). Together with histone-modifying enzymes, Nrf2 recruits Mediator, a multi-subunit protein complex that communicates the activation signals from a DNA-bound transcription factor to RNA polymerase II (Pol II) (6, 143). The subunit composition of Mediator can change: subunits can be lost or added to affect its biological function (6). NRF2 has been shown to interact directly with the MED16 subunit of Mediator though the Neh1, Neh4, and Neh5 domains (143). MED16 bridges the interaction between NRF2 and the Mediator complex and its depletion specifically reduces the transcription of several NRF2 target genes in response to electrophilic stress, without affecting hypoxia-induced gene expression. *MED16* knockout does not impact cell proliferation but renders the cells more sensitive to cytotoxicity induced by menadione (143). In addition to histone-modifying enzymes and the Mediator complex, NRF2 recruits ATP-dependent nucleosome-remodeling complexes. BRG1, the central ATPase subunit of the SWI/SNF chromatin-remodeling complex, has been shown to interact with NRF2 and to selectively influence the transcription of NRF2 target genes with Z-DNA formation (193). Moreover, NRF2 associates with other co-activators of the transcription machinery, such as chromodomain helicase DNA-binding protein 6 (CHD6) (120), receptor-associated co-activator 3 (RAC3) (75), and NAD^+^-dependent histone deacetylase sirtuin 6 (SIRT6) (128). However, the functional significance of these interactions has not been extensively elucidated.

Together, co-activators and chromatin remodelers promote the recruitment of Pol II and the components of the general transcription machinery to form the pre-initiation complex (PIC) at the promoters of target genes. Following PIC assembly, the carboxy-terminal domain (CTD) of Pol II has to be phosphorylated at Ser5 to initiate transcription of the gene and subsequently at Ser2 to promote productive transcription elongation (179). In *Drosophila*, cyclin-dependent kinase 12 (Cdk12) has been identified as the Pol II kinase responsible for the phosphorylation of Ser2 in Pol II CTD at Nrf2 target genes on exposure to oxidative stress. Under unstressed conditions, knockdown of *Cdk12* in cell culture and *in vivo* does not affect cell viability or the expression of genes involved in basic housekeeping processes; however, in response to the oxidative stress inducer paraquat, it specifically impairs the expression of Nrf2 target genes and decreases the survival of the flies (93). In human cells, CDK12 has been identified as an essential regulator for the transcription of various DNA damage response and DNA repair genes, increasing the interest in the development of pharmacological inhibitors of CDK12 to act as sensitizers to chemotherapeutic agents (64, 94). If Cdk12 gene selectivity for Nrf2 antioxidant targets is conserved in humans, the mechanism of action of Cdk12 inhibitors may not be limited to the repression of the DNA damage response and DNA repair pathway, but may also involve the suppression of Nrf2 cytoprotective response.

## Deciphering the Nrf2-Regulated Network

### Mapping of the Nrf2 transcriptional program

Continuous efforts have been made to identify the genes regulated by Nrf2 and thus the function of Nrf2 in a given cellular context. These studies have utilized pharmacological activation of Nrf2, *Nfe2l2*-deficient or *Keap1*-deficient mice to define the Nrf2 responsive genes. The first of these studies performed gene expression profiling by microarray of the small intestine from wild-type and *Nfe2l2* knockout mice treated with vehicle or the Nrf2 inducer SFN and identified that the basal and inducible expression of several genes involved in ROS detoxification, GSH synthesis and NADPH regeneration is dependent on Nrf2 (164).

Transcriptional profiles of *Nfe2l2* knockout mice showed that Nrf2 is not just involved in inducible gene expression in response to an activating agent but also involved in the constitutive expression of several antioxidant and detoxification genes in the absence of external stresses (13, 100, 164). Under normal conditions, Nrf2 is a very unstable protein with a short half-life; therefore, it was surprising to observe Nrf2-mediated regulation of gene expression in the absence of exogenous stress stimuli (118, 156). Since ROS and other endogenous reactive molecules are constantly generated from physiological cellular processes, it is possible that Keap1 activity is slightly impaired under unperturbed conditions. As discussed above, Nrf2 can also be regulated at the transcriptional level (49). Moderate Nrf2 activation in the absence of external stresses is likely the result of equilibrium between *Nfe2l2* transcription and protein stability.

These gene expression analyses were performed in several tissues and used different agents to induce Nrf2, including naturally occurring chemopreventive drugs, such as SFN, soy isoflavone and triterpenoids, and toxicants, such as hypochlorous acid and inorganic arsenite, and showed that Nrf2 regulates a common set of genes irrespective of the type of stimulus and the cellular context (13, 27, 164, 182, 188). This “default Nrf2 program” includes genes such as *Nqo1*, *Gclc*, *Gclm*, and *Txnrd1* and is in part conserved from *Drosophila* to humans, constituting an ancient Nrf2 regulatory network (89). Together with the “default Nrf2 program,” Nrf2 selectively activates other genes that are specific to the cell type and the nature of the inducing agent (2, 13, 82, 89, 107, 164, 182, 188).

The current studies of the Nrf2-dependent program are limited to the investigation of the response to pharmacological activation of Nrf2 or to the deletion of either *Keap1* or *Nfe2l2* in different cellular contexts. Studies comparing how the Nrf2-dependent network changes from a physiological to a pathological condition are still lacking. The only exception is represented by a work on the mechanism of Nrf2-mediated oncogenicity in lung cancer (142). Transcriptional profile in normal lung tissue and urethane-induced tumors from wild-type and *Nfe2l2* knockout mice showed induced expression of genes involved in cell growth and proliferation, Wnt/β-catenin signaling, and Notch signaling in an Nrf2-dependent manner, suggesting a possible mechanism for the contribution of Nrf2 to cancer progression (142).

### Nrf2-mediated gene repression

These large-scale studies indicated that Nrf2 not only activates but also suppresses the expression of a wide range of targets. However, the mechanisms underlying transcriptional repression by Nrf2 are still unclear. Gene expression profiling by microarray in mouse liver identified genes that were repressed by H-1,2-dithiole-3-thione (D3T) in wild-type mice, but not in *Nfe2l2*-knockout mice, compared with vehicle-treated wild-type mice. Of note, these downregulated genes were not detected at the early time point (6 h) but only at the late time point (24 h), suggesting that the repression observed might be indirect (88). In line with this observation, the integrated analysis of ChIP-Seq and RNA-Seq data showed that the majority of Nrf2 direct targets are upregulated rather than repressed, indicating that Nrf2 is primarily an activator and blocks gene expression indirectly (23, 52) (C.T., I.I.C.C. and D.A.T. unpublished observations). These data contradicted previous studies, which reported that several transcription factors could form inhibitory complexes with Nrf2 and bind the promoters of Nrf2 target genes, thus causing their transcriptional repression (8, 15, 57). Nrf2 may repress transcription indirectly by inducing the expression of transcriptional repressors or miRNAs (144). NRF2 ChIP-Seq in lymphoblastoid cell lines identified several NRF2 binding sites in the vicinity of multiple miRNAs, the most notable being the miR-365-1/miR-193b cluster and miR-29b-1, which were previously linked to cancer progression and oxidative stress, respectively (23). An additional example of Nrf2-controlled miRNA is miR-125-b1, which is upregulated after activation of Nrf2 in the kidney of mice treated with oltipraz (65).

### Nrf2 distal binding sites

The global mapping of Nrf2 binding sites indicated that the majority of Nrf2 binding sites lie outside the promoter-proximal region (23, 52). Nrf2 function at distal genomic sites is still unclear. Some of these binding sites could be located in the promoter region of long non-coding RNAs (lncRNAs), which were beginning to be annotated when Nrf2 binding sites were first profiled (44). To date, few Nrf2-regulated lncRNAs have been described. NRF2 was shown to activate *SCAL1* lncRNA in response to cigarette smoke in lung cancer cell lines (162) and to repress the pluripotency lncRNA *ROR* in human MCF10A mammary epithelial cells (195). Furthermore, a recent study identified additional NRF2-regulated lncRNAs by transcriptomic analysis of tumors with activating mutations in *NFE2L2* and validated *LINC00942* as a new Nrf2 direct target involved in modulating *GCLC* expression through an unclear mechanism (9). The profiling of Nrf2-dependent genes has been mainly performed by microarray analysis, which usually does not allow the detection of lncRNAs. With the transition to deep sequencing approaches for measuring gene expression, the list of Nrf2-regulated lncRNAs will continue to grow.

In addition, distal Nrf2 binding sites could be located at enhancers. Enhancers are genomic domains that regulate transcription by functioning as binding platforms for transcription factors and are characterized by specific chromatin signatures of histone methylation and acetylation (135). The generation of chromatin state maps by profiling combinations of epigenetic marks, in addition to Nrf2 in a given cell type will allow the identification of noncoding regulatory elements bound by Nrf2 and provide unique insights into Nrf2-mediated transcriptional regulation. The investigation of the relationship between Nrf2 binding and the dynamics of the local chromatin environment will also inform on Nrf2 activity at those sites.

The systematic characterization of NRF2-bound regulatory elements becomes particularly relevant in light of evidence for positive selection of SNPs at specific NRF2 binding sites that could influence gene expression and, ultimately, disease risk (105, 178).

### Crosstalk between Nrf2 and other signaling pathways

Analyses of transcriptional responses have revealed complex interactions between the Nrf2 regulatory network and other signaling cascades. As previously discussed, Nrf2 activity can be modulated in multiple ways by several signaling pathways, thereby affecting the expression of Nrf2 target genes. Conversely, Nrf2 can both positively and negatively influence the downstream pathways of other transcription factors and these interconnections can occur in various forms. For example, Nrf2 can regulate the expression or the stability of other transcriptional regulators. Gene expression microarray analyses comparing *Nfe2l2*-null and wild-type MEFs showed that expression of *Notch1* and *AhR*, as well as their target genes, is decreased in *Nfe2l2*-depleted cells (148, 172). In addition, a study on *Nfe2l2*-null MEFs revealed enhanced IκB kinase β activity, which phosphorylates IκB, the negative regulator of NF-κB, thus inducing IκB degradation and NF-κB activation (163).

Moreover, several Nrf2 inducing agents can concomitantly trigger the activation of other transcriptional regulators, thus creating complex interconnections between their signaling pathways (3, 51, 70, 154). For example, Nrf2 and AhR have been shown to collaborate to mediate the response to 2,3,7,8-tetrachlorodibenzo-p-dioxin, 3-methylchoranthrene, butylated hydroxyanisole, and phenobarbital (98, 121, 189). In addition, disruption of Nrf2 delayed liver regeneration after partial hepatectomy and this phenotype was rescued by expression of the Notch1 intracellular domain, suggesting a functional crosstalk between the Nrf2 and Notch1 pathways (172). It is noteworthy that AhR, Notch, and NF-κB can regulate Nrf2 expression, indicating bidirectional interactions between these pathways (138, 148, 173).

Finally, Nrf2 activity can be modulated through cross-binding with other transcription factors. In cancer cells, mutant p53 has been shown to piggyback on NRF2 to regulate the expression of proteasome genes, leading to resistance to the proteasome inhibitor carfilzomib (174).

A more comprehensive elucidation of the crosstalk between Nrf2 and other signaling pathways will help to decipher the complexity of Nrf2-driven cellular processes. In an attempt to dissect the Nrf2 interactome and regulome, Korcsmáros and colleagues developed NRF2-ome, an integrated web resource containing information on Nrf2 interacting factors, target genes, regulating transcription factors, and miRNAs (129, 168). It will be important to extend the current computational and experimental approaches to obtain a more dynamic global view of Nrf2-mediated gene regulation that integrates all the factors that influence the final transcriptional output.

## Conclusions and Perspectives

**FIG. 6.**
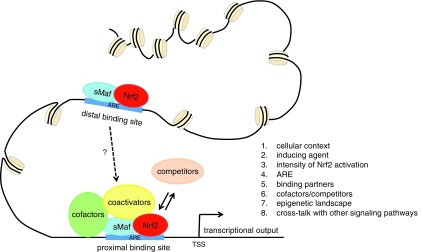
**Dissecting the Nrf2 network.** Nrf2 lies at the center of a complex regulatory network. Nrf2-mediated regulation of gene expression depends on the cellular context, the inducing agent, the levels of Nrf2 activation, the recognition of the ARE, binding partners, cofactors and competitors, the crosstalk with other signaling pathways, and the epigenetic landscape of the target gene promoter, among others.

Since the discovery of Nrf2 in 1994 (108), our understanding of its biology has continued to grow. Nrf2 has been implicated in different cellular processes, such as the response to oxidative and xenobiotic stress, mitochondrial respiration, stem cell quiescence, mRNA translation, autophagy and UPR. Significant advances have been made in understanding the regulation of Nrf2 activity, downstream pathways and implications for the development of disease. It is now clear that Nrf2 lies at the center of a complex regulatory network. Nrf2-mediated transcriptional regulation is determined by the cellular context, the activating stimulus, the recognition of the ARE, the availability of binding partners, the competition or cooperation with other activators and repressors, the crosstalk with other signaling pathways and the epigenetic landscape of the target gene promoters, among others (Fig. 6). A complete appreciation of the Nrf2 program will require an integrated consideration of all these factors, which will allow these efforts to have the most profound benefits to human health.

## Abbreviations Used

β-TrCP: β-transducing repeat-containing protein
ABSC: airway basal stem cell
AhR: aryl hydrocarbon receptor
AML: acute myeloid leukemia
AP1: activator protein 1
ARE: antioxidant response element
ATF4: activating transcription factor 4
Bach1: BTB domain and CNC homolog 1
Bach2: BTB domain and CNC homolog 2
bZIP: basic-region leucine zipper
BRCA1: breast cancer susceptibility 1
CBP: CREB binding protein
Cdk: cyclin-dependent kinase
ChIP-Seq: chromatin immunoprecipitation followed by sequencing
CNC: cap ‘n’ collar
CncC: cap ‘n’ collar isoform C
CTD: carboxy-terminal domain
ER: endoplasmic reticulum
Gcl: glutamate-cysteine ligase
Gclc: glutamate-cysteine ligase catalytic subunit
Gclm: glutamate-cysteine ligase modifier subunit
Gpx2: glutathione peroxidase 2
GSH: glutathione
Gsk-3β: glycogen synthase kinase-3β
GSSG: oxidized glutathione
Gst: glutathione S-transferase
HbSS: homozygous sickle cell disease
HGPS: Hutchinson-Gilford progeria syndrome
Hmox1: heme oxygenase 1
Hrd1: synoviolin
Idh1: isocitrate dehydrogenase 1
ISC: intestinal stem cell
Keap1: Kelch-like ECH-associated protein 1
lncRNAs: long non-coding RNAs
MEF: mouse embryonic fibroblast
miRNA: microRNA
NADPH: nicotinamide adenine dinucleotide phosphate
Neh: Nrf2-ECH homology
NF-E2: nuclear factor, erythroid-derived 2
NF-κB: nuclear factor-κB
Nqo1: NADPH quinone dehydrogenase 1
Nrf2: nuclear factor E2-related factor 2
Pgd: 6-phosphogluconate dehydrogenase
PI3K: phosphoinositide 3-kinase
PIC: pre-initiation complex
PKC: protein kinase C
Pol II: RNA polymerase II
PPP: pentose phosphate pathway
ROS: reactive oxygen species
RXRα: retinoid X receptor α
SCF: Skp1-Cul1-F-box
SFN: sulforaphane
sMaf: small masculoaponeurotic fibrosarcoma
SNP: single nucleotide polymorphism
TXN: thioredoxin
Txnrd1: thioredoxin reductase 1
UPR: unfolded protein response
